# Novel methylation mark and essential hypertension

**DOI:** 10.1186/s43141-022-00301-y

**Published:** 2022-01-21

**Authors:** Mayank Chaudhary

**Affiliations:** Department of Biotechnology, Maharishi Markandeshwar (Deemed to be) University, Mullana-Ambala, Haryana 133207 India

**Keywords:** Essential hypertension, 5-methylcytosine, 6-methyladenine, Epigenetics, DNA methylation, Methyltransferase, Demethylase

## Abstract

**Background:**

Essential hypertension (EH) is an important risk factor for various cardiovascular, cerebral and renal disorders. It is a multi-factorial trait which occurs through complex interplay between genetic, epigenetic, and environmental factors. Even after advancement of technology and deciphering the involvement of multiple signalling pathways in blood pressure regulation, it still remains as a huge global concern.

**Main body of the abstract:**

Genome-wide association studies (GWAS) have revealed EH-associated genetic variants but these solely cannot explain the variability in blood pressure indicating the involvement of additional factors. The etiopathogenesis of hypertension has now advanced to the level of epigenomics where aberrant DNA methylation is the most defined epigenetic mechanism to be involved in gene regulation. Though role of DNA methylation in cancer and other mechanisms is deeply studied but this mechanism is in infancy in relation to hypertension. Generally, 5-methylcytosine (5mC) levels are being targeted at both individual gene and global level to find association with the disease. But recently, with advanced sequencing techniques another methylation mark, N6-methyladenine (6mA) was found and studied in humans which was earlier considered to be absent in case of eukaryotes. Relation of aberrant 6mA levels with cancer and stem cell fate has drawn attention to target 6mA levels with hypertension too.

**Conclusion:**

Recent studies targeting hypertension has suggested 6mA levels as novel marker and its demethylase, ALKBH1 as probable therapeutic target to prevent hypertension through epigenetic programming. This review compiles different methylation studies and suggests targeting of both 5mC and 6mA levels to cover role of methylation in hypertension in broader scenario.

## Background

**Fig. 1:**
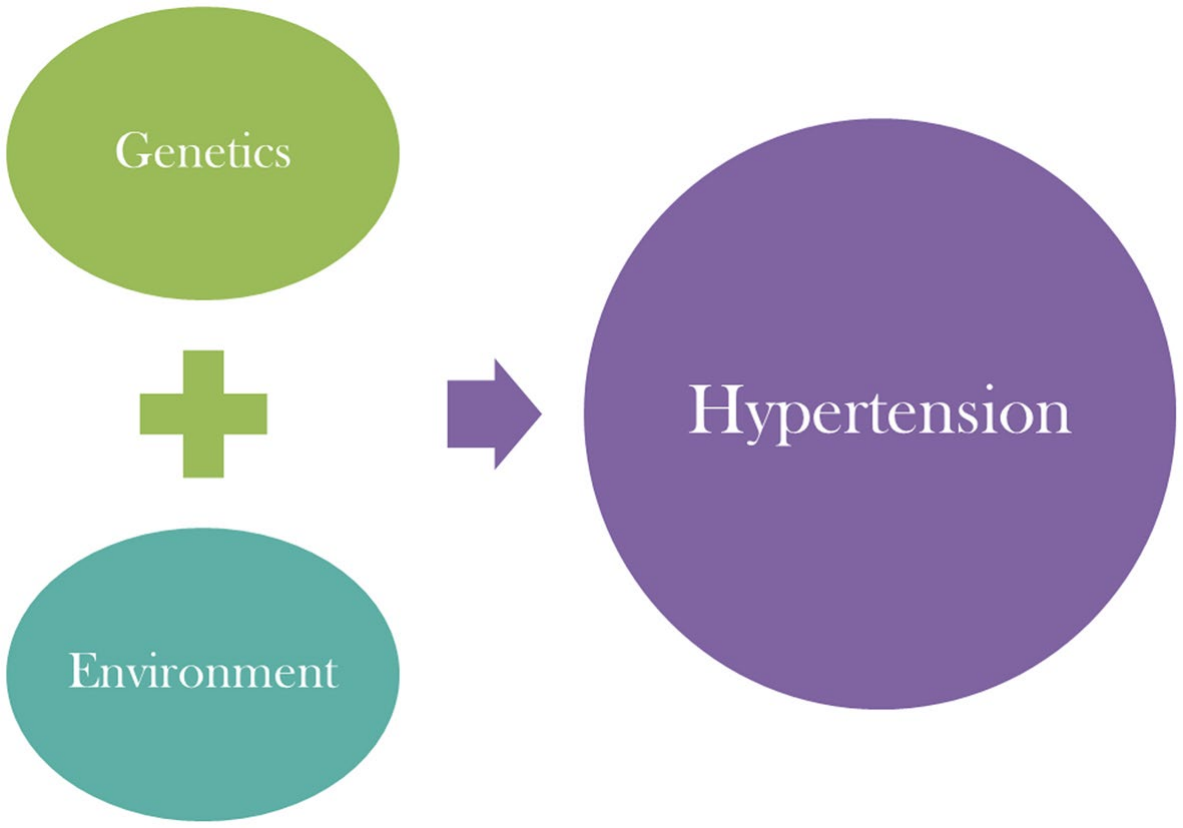
Gene-environment interaction for phenotypic manifestation of hypertension

**Fig. 2:**
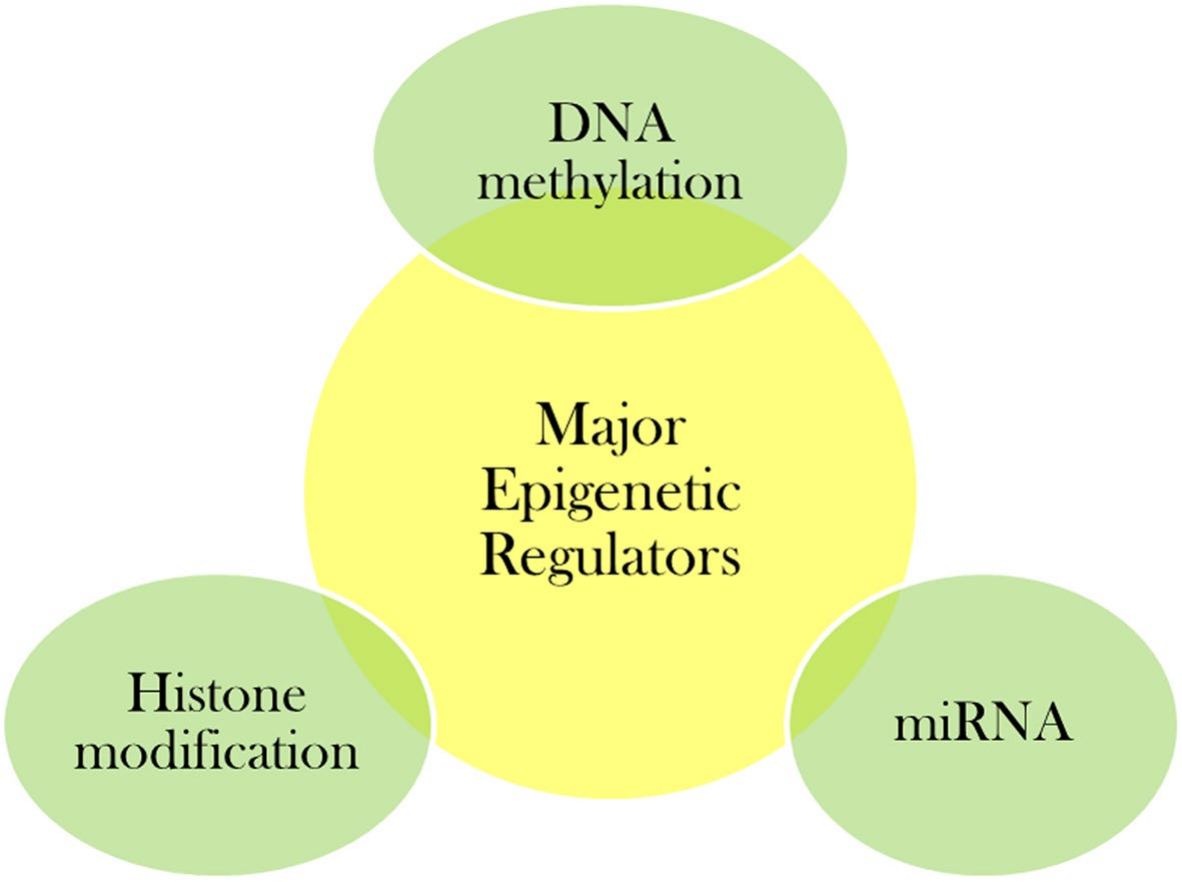
Major epigenetic regulators

Hypertension, more commonly known as high blood pressure, occurs due to constantly high pressure on the walls of blood vessels. It is broadly classified into primary and secondary where primary or essential hypertension (EH) is more prevalent form affecting around 95% of adult patients having high blood pressure [1]. Primary hypertension is one which occurs without any known identifiable cause whereas secondary hypertension occurs secondarily to certain other primary ailment. Hypertension affects more than 1.5 billion people worldwide and is an important risk factor for various cardiovascular, cerebrovascular and other renal disorders causing considerable public health concern [2]. EH is a complex disorder [3] and occurs as a result of interplay between genetic and environmental factors (Fig. 1). Though association studies [4–6] targeting specific genes have found certain polymorphisms to be linked with the pathogenesis of hypertension but such association studies sometimes culminates into non-reproducible results in another population cohort. Additionally, genome-sequencing results have revealed presence of disease causing mutation without phenotypic presence [7]. Genome-wide association studies (GWAS) have identified different genetic loci to be associated with hypertension [8–11]. These genetic loci accounts for small fraction of heritability highlighting either the cumulative effect of such polymorphisms or role of some other non-genetic factor (epigenetic mechanisms) that can be modified through lifestyle or environmental factors [1] for disease outcome. This led to epigenome-wide association studies (EWAS) where DNA methylation at different CpG sites was found to be associated with blood pressure in individuals from varied ancestry [12, 13]. Epigenetics refer to the changes that occur in gene expression pattern without any change in the DNA sequence. Epigenetic modifications can be initiated by different factors that include environmental influence during foetal development, dietary habits, routine lifestyle, aging, and sometimes medical prescriptions [14]. Most commonly targeted epigenetic modifications (Fig. 2) include DNA methylation, histone modification and RNA-based mechanisms [15]. Out of these, DNA methylation is the vastly studied epigenetic mechanism affecting gene regulation in mammals [16]. It is best characterized epigenetic mark in relation to cancer but its role in hypertension is still in its infancy. Readers are suggested for detailed reviews [3, 17–19] on epigenetic mechanisms targeting blood pressure regulation as current article focuses specifically on the role of DNA methylation with emphasis on novel methylation mark in relation to hypertension.

## Main text

### DNA methylation

**Fig. 3:**
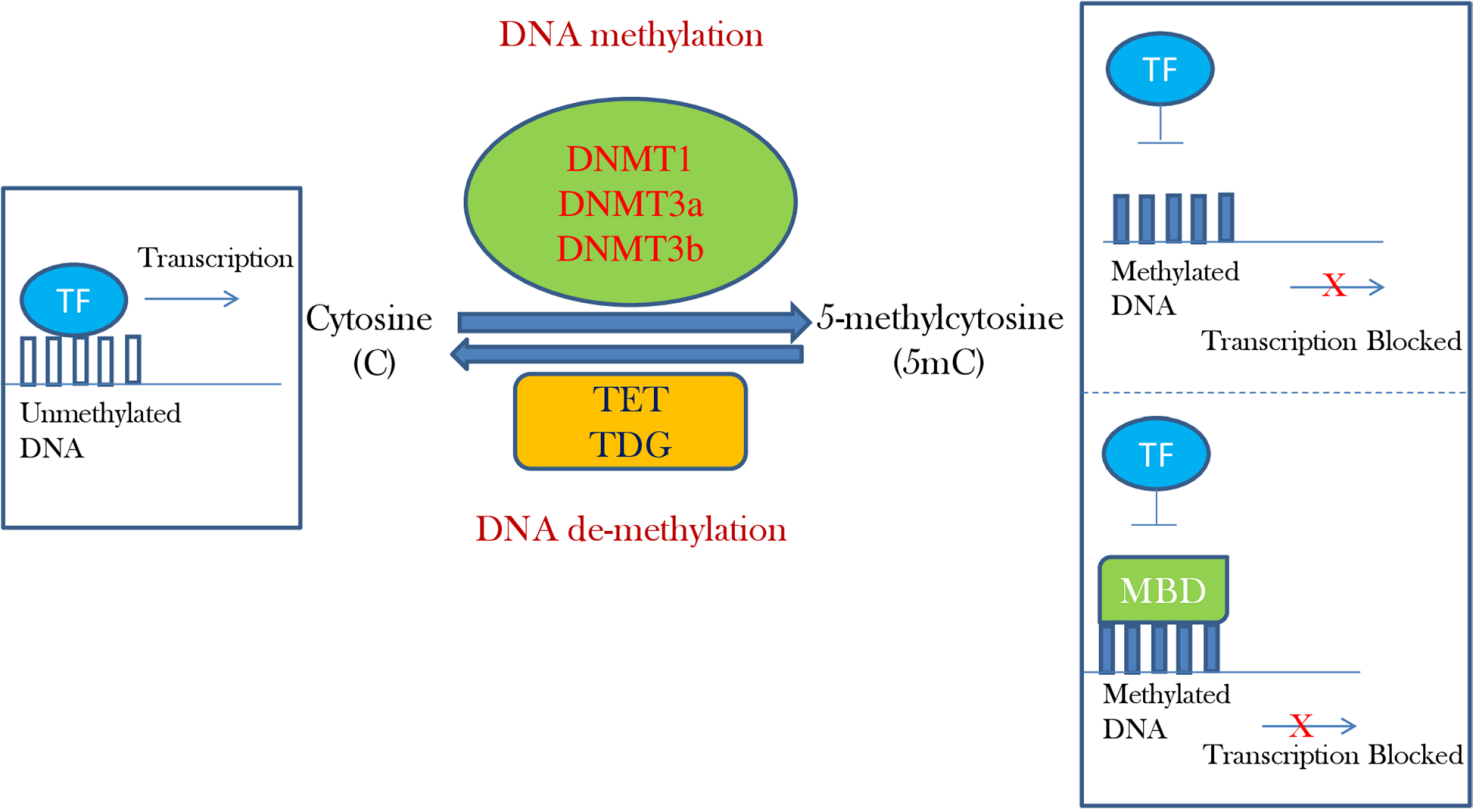
Role of DNA methylation on gene expression

DNA methylation plays a crucial role in regulating many cellular processes like embryonic development, X-chromosome inactivation (inactivation of one of the female X-chromosome for dosage compensation), and genomic imprinting (epigenetic marks control gene expression in parent-specific manner where either of paternally or maternally inherited allele is active rather than activation of both alleles) [20]. DNA methylation in mammals mainly occurs at cytosine in cytosine-guanine dinucleotides (CpG) which involves the transfer of methyl group (CH_3_) from S-adenosyl-methionine (SAM) to the 5 carbon position of cytosine resulting in the formation of 5-methylcytosine (5mC) [15]. The “P” identifies the presence of phosphodiester bond between cytosine and guanine nucleotides [21]. CpG islands are mostly located within the promoter region or in the end 5′ region and are generally unmethylated [22]. CpG dinucleotide sites other than the promoter region have different methylation status and are methylated [23]. Methylation profile of cytosine in promoter CpG islands affects expression pattern of the gene. Promoters can be differentiated as high, intermediate, and low CpG promoters and anti-correlation has been found between CpG density and DNA methylation in genome wide DNA methylation analysis [24]. DNA methylation is functionally linked to suppression of gene transcription [14] (Fig. 3). This is achieved either directly by preventing the binding of transcriptional activators to promoter region or indirectly by attachment of methyl-CpG binding proteins (MBP) [25]. Mammalian DNA methylation is carried by DNA methyltransferases (DNMTs) including DNMT1, DNMT3a, and DNMT3b (Fig. 3). DNMT1 is mainly responsible for maintaining DNA methylation pattern in the newly synthesized DNA strand whereas DNMT3a and DNMT3b are responsible for de novo DNA methylation [26]. Pathway for active DNA demethylation involves ten-eleven-translocases (TET) family (Fig. 3) of proteins [27] that converts 5mC to 5-hydroxymethlycytosine (5hmC) and further to 5-formylcytosine (5fC) which subsequently is oxidised to form 5-carboxylcytosine (5caC) [1, 28]. These TET dependant oxidation products are considered as demethylation intermediates of 5mC playing crucial role in transcription, chromatin remodelling and DNA repair [29]. Both 5fC and 5caC are recognized and excised by thymine-DNA glycosylase (TDG) resulting in replacement by unmodified cytosine [1]. Effect of DNA methylation on gene expression is vastly studied in relation to cancer but its role with context to hypertension has recently gained interest. Pioneer work on rats highlighted the role of DNA methylation on blood pressure regulating genes.

### DNA methylation and hypertension

One of the pioneer studies targeting the effect of diet on blood pressure was done by Bogdarina et al. [30]. Low protein diet in pregnant rats yielded hypertensive offsprings by upregulation of *AT1b* gene through promoter hypomethylation. Similarly, maternal low protein diet (MLPD) altered expression of RAS genes in fetal brains where promoter hypomethylation increased expression of *ACE* gene [31]. In similar manner, MLPD during gestation led to hypertension in sexually dimorphic pattern in the offsprings where females were severely affected and males were moderately hypertensive [32]. Further studies on rats showed enhanced expression of *AT1a* in offsprings of nicotine administered pregnant rats as a result of promoter hypomethylation resulting in hypertension [33]. Promoter hypomethylation heightened *AT1a* expression causing increased blood pressure in spontaneously hypertensive rats (SHR) compared to Wistar-Kyoto (WKY) rats [34]. Similarly, hypomethylation-related upregulation of Na-K-Cl cotransporter I gene (*NKCC1*) caused hypertension in SHR [35]. Riviere et al. [36] performed in vitro and in vivo studies targeting the role of DNA methylation on promoter CpG islands of *ACE* gene. Recently epigenetic studies on human genes regulating blood pressure have also come up where methylation status at global and individual gene level is being targeted. Lower 5mC levels in DNA of hypertensive individuals was found by Smolarek et al. [37] whereas increased global DNA methylation levels were found in pre-eclampsia group by Kulkarni et al. [38] in comparison to controls. Genome-wide methylation analysis validated significant CpG sites in sulfatase gene (*SULF1*) and prolylcarboxypeptidase gene (*PRCP*) [39]. Methylation analysis between cases and controls showed significant methylation difference in the promoter CpG site of *SULF1* gene where hypermethylation was observed in the hypertensive cases. Similarly, trans-ancestry genome wide association on East Asian, European, and South Asian individuals found genetic variants at 12 new loci in the genes involved in vascular smooth muscle and renal function to be associated with blood pressure [40]. Sentinel blood pressure SNPs from this study were found to be affected by methylation status of nearby CpG sites highlighting the role of DNA methylation on blood pressure regulation. Along with such studies on global methylation difference, role of DNA methylation pattern in altering gene expression is also studied at individual gene level. Enzyme 11 beta-hydroxysteroid dehydrogenase type 2 (11 beta-HSD2) is involved in the conversion of cortisol to cortisone and inactivity of this enzyme results in cortisol-based activation of mineralocorticoid receptors resulting in sodium retention and elevated blood pressure. Study on epigenetic control of *HSD11B2* gene coding for this enzyme showed that promoter methylation resulted in hypertension by gene downregulation [41]. Promoter hypomethylation at 5 CpG sites in the promoter of alpha-adducin gene (*ADD1*) coding for α-adducin was found to be associated with hypertension. Gender-based segregation related hypomethylation at CpG1 with increased risk of EH in females whereas hypomethylation at CpG2-5 sites increased the risk in males [42]. Similarly, methylation pattern in CpG island of glucokinase (*GCK*) gene showed association with EH. Patients with EH showed hypomethylation at CpG1-3 sites and hypermethylation at CpG4 site of the studied gene [43]. Additionally, genes in which role of promoter methylation in EH is targeted includes angiotensin II type 1 receptor gene, *AGTR1* [44, 45], aldosterone synthase gene-Cytochrome P450 family 11 subfamily B member 2, *CYP11B2* [46], sodium channel epithelial 1 beta subunit gene, *SCNN1B* [47], interleukin-6 gene, *IL-6* [48], toll-like receptor 2 gene, *TLR2* [49], and mitofusin 2 gene, *Mfn2* [50]. Association between promoter hypermethylation of beta-3-adrenoreceptor gene (*ADRB3*) with dyslipidemia and blood pressure was targeted by Guay et al. [51].

### N6-methyladenine as new epigenetic mark

**Fig. 4:**
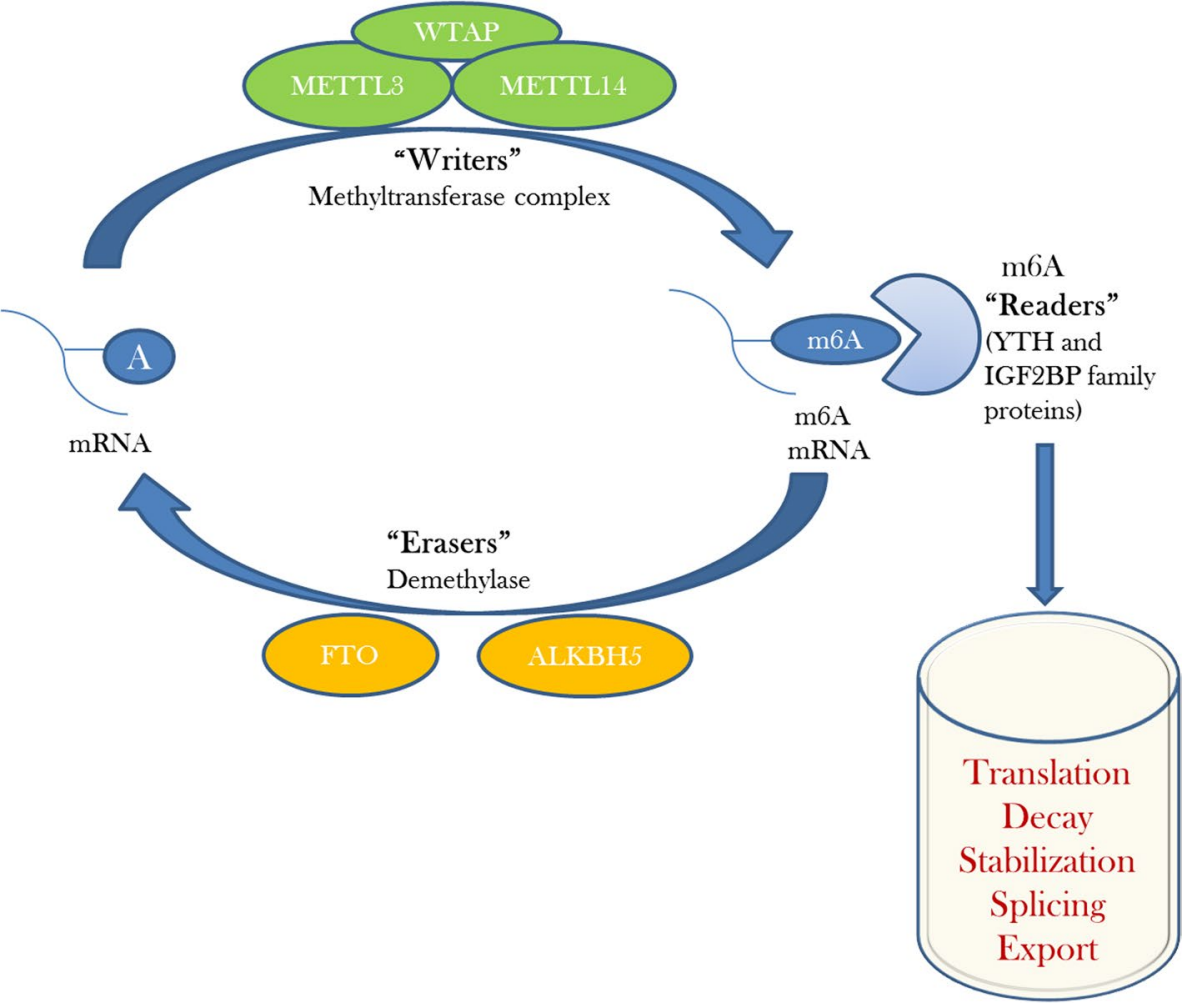
Regulation of m6A modification and role in RNA processing

Three type of methylated adenine bases are considered biologically relevant where 1-methyladenine (1mA) and 3-methyladenine (3mA) occurs due to alkylation damage in DNA and 6-methyladenine (6mA) is mainly attributed by enzymatic activity [52]. 6mA and m6A refers to methylated adenine in DNA and RNA respectively. In contrast to 5-methylcytosine (5mC), N6-methyladenine (6mA) modification of DNA is more prevalent in case of prokaryotes where it plays crucial role from DNA replication and repair to viability of certain bacterial strains [53–55]. Earlier studies failed to detect DNA 6mA modification in eukaryotes which led to consideration of absence of such modification in the eukaryotic system including humans [56]. But with the technological breakthroughs and development of deep sequencing, eukaryotic species were also reported to possess 6mA DNA modification including *Chlamydomonas reinhardti*, *Caenorhabditis elegans*, *Drosophila melanogaster*, *Arabidopsis thaliana*, fungi, *Mus musculus*, *Oryza sativa*, zebra fish, and pig [53, 57, 58]. In addition to other chemical modifications of RNA, m6A RNA modification of human mRNA plays pivotal role in RNA splicing, mRNA stability and gene expression [53, 57]. Presence of 6mA DNA modification was considered to be absent in humans in previous studies [56] but advanced sequencing techniques found presence of 6mA even in human genomic DNA [59]. 6mA DNA modification sites were identified in the human genome which accounted for approximately 0.051% of the total adenines in the human genome and results indicated [G/C]AGG[C/T] as most significant motif associated with 6mA modification [53]. This group further presented data on density distribution of 6mA across all human chromosomes and found presence of 6mA modification sites mostly in the intronic and intergenic regions with no significant enrichment of 6mA around transcription start sites (TSSs) and transcription terminal sites (TTSs) in the human genome [53]. The functional effects of N6-methyladenine modification is regulated by methyltransferases, demethylases and binding proteins [55]. Methyltransferase responsible for human 6mA DNA modification is N-6 adenine-specific DNA methyltransferase 1 (N6AMT1) as observed through silencing and overexpression experiments [53]. Similarly, methyltransferase-like 3 (METTL3) and methyltransferase-like 14 (METTL14) methyltransferase complex in association with adaptor proteins like Wilms’ tumor 1 associating-protein (WTAP), RNA binding protein motif 15 (RBM15) and KIAA1429 is responsible for m6A modification where adaptor proteins contribute to functional activity of methyltransferase complex [55, 60, 61]. m6A RNA readers recognize and bind to m6A sites in RNA regulating downstream functions by affecting splicing, export, stability, and translation of mRNA [55, 62, 63]. These m6A RNA readers include YTH family of proteins comprising of YTHDC1, YTHDC2 and YTHDF (YTHDF1, YTHDF2, and YTHDF3) family and IGF2BP family proteins (IGF2BP1, IGF2BP2, and IGF2BP3) [55, 64]. YTH family of proteins recognize through YTH RNA-binding domain [55, 65] and IGF2BP family proteins recognize and bind GG (m6A)C sequence through K homology domains [55]. Demethylation of DNA 6mA is performed by ALKBH1 enzyme [53–55] whereas demethylases involved in removal of m6A methylation include fat mass and obesity-associated protein (FTO) and AlkB homolog 5 (ALKBH5) [55, 57, 61] (Fig. 4). Involvement of m6A modification is found in circadian rhythm, viral replication, embryogenesis and development of human diseases (obesity, cancer and neuronal disorders) [58, 61, 62]. Recent data have shown involvement of DNA 6mA modification in the development and progression of cancer. Increased activity of demethylase AKLBH1 and decreased activity of N6AMT1 methyltransferase was reported in cancer cells that resulted in decreased abundance of 6mA in targeted cancer tissues [53]. Contrary to this, higher levels of DNA 6mA were observed in glioblastoma stem cell (GSC) and primary glioblastoma specimens compared to normal human astrocytes relating higher 6mA levels with disease progression [66]. Another major role of DNA 6mA modification was related with DNA damage repair [67] where N6-methyladenine minimizes incorporation of 8-oxo-2’-deoxyguanosine (8-oxoG) against adenine by DNA polymerase. Thereby, preventing the chance of mutation through oxidative damage by avoiding mispairing of 8-oxoG with adenine. Additionally, the role of m6A modification in regulating fate of stem cell was also reported [68]. Depletion of ALKBH1 demethylase in mice resulted in deformities in eyes, skeletal and neural system and even early embryonic lethality [69–71]. Knockout of *ALKBH1* gene resulted in undifferentiated state of embryonic stem cells (ESCs) suggesting possible role of m6A in transcriptional silencing [72]. Contradictory results of m6A level on stem cell fate also exists where inhibition of METTL3 methyltransferase complex in embryos of zebrafish prevented progression of cell cycle and resulted in developmental defects [73].

### N6-methyladenine modification and hypertension

Role of novel DNA methylation modification, N6-methyladenine (6mA) in the prognosis of hypertension is largely unclear. Recently, handful of papers has found relation between 6mA DNA level and hypertension. Reduced 6mA methylation level was found in the leukocytes of hypertensive patients which returned back to normal after successful treatment of hypertension [74]. Results similar to clinical investigation were observed by them in both mouse and rat hypertension models which showed reduced 6mA DNA methylation level in the leukocytes. Increased ALKBH1 level reduced global 6mA DNA level in human aortic smooth muscle cells (HASMCs) treated with different concentrations of Ang II and endothelin 1 (ET-1). Results were confirmed through siRNA transfection where silencing of ALKBH1 reduced the reduction in 6mA DNA level in the treated HASMCs [74]. Similarly, elevated AKLBH1 level was found to be associated with reduced 6mA DNA level in the vascular smooth muscle cells (VSMCs) of hypertension models through in vitro and in vivo studies. Further experiments identified hypoxia inducible factor 1 α (H1F1α) that is required for Ang II-induced vascular remodelling as the target of ALKBH1. Relation between them was found as elevation in H1F1α level was inhibited by silencing of ALKBH1. Their findings suggested 6mA as sensitive marker for diagnosis of hypertension and ALKBH1 as novel therapeutic target to prevent hypertension through epigenetic reprogramming because ALKBH1 mediates cross talk between 6mA DNA level and H1F1α activity. In contrary to this, earlier reports found increased m6A levels due to decreased expressed or mutations of another demethylase gene, *FTO* to be associated with hypertension and cardiovascular diseases [75, 76]. On the basis of these results, Paramasivan et al. [77] suggested targeting of m6A levels through methyltransferase (METTL3) and demethylase (FTO) enzyme as potential therapeutic strategy for hypertension. Pulmonary artery hypertension (PAH) disease is characterized by pulmonary vascular remodelling culminating in right heart failure [78]. Role of methylation in disease intervention of PAH has been observed [79] but the role of internal modification of mRNA, N6-methyladenine (m6A) in PAH was recently studied [64]. Experiments were performed in mouse model with SET domain containing 2 histone lysine methyltransferase (*SETD2*)-specific knockout of smooth muscle cells (SMCs). SETD2 enzyme catalyzes trimethylation of lysine 36 on histone 3 (H3K36me3) which is a histone marker for active transcription region [80]. Lack of SETD2 in SMCs protected mice from hypoxia-induced PAH and further reduced level of methyltransferase like 14 (METTL14) and methylation of m6A RNA [64]. Involvement of SETD2-mediated H3K36me3 in PAH was observed where clear differences were found in protein levels of SETD2 and H3K36me3 in SMCs and SETD2-deficient SMCs. SETD2-deficient SMCs further showed impaired protein level of METTL14 and diminished m6A RNA levels [64]. Their results demonstrated that SETD2 mediated m6A RNA modification through H3K36me3 and METTL14 to result into hypoxia-induced PAH in mice. They further suggested inhibition of SETD2/METTL14 activity as a possible strategy for treatment of pulmonary artery hypertension in clinical settings. These few ongoing studies provides a hint towards potential role of 6mA level in the progression of hypertension which can be targeted in future studies deciphering the role of methylation in disease manifestation.

## Conclusion

N6-methyladenine modification of DNA is prominently found in prokaryotes but considered to be absent in case of eukaryotes. With the development of deep sequencing it was found to be present in limited number of eukaryotes too. Though m6A modification of mRNA plays crucial role in RNA splicing, export, stability, decay, and gene expression in humans but presence of 6mA in human genomic DNA was recently identified through single-molecule real-time (SMRT) sequencing technology. Role of methyltransferase and demethylase responsible for affecting 6mA and m6A levels was recently found in different diseases and mechanisms involving cancer and stem cell fate. As a result, research targeting role of these enzymes in hypertension found relation of 6mA level with hypertension progression. Current research suggests targeting of 6mA level as novel diagnostic marker and demethylases, AKLBH1, and FTO as potential therapeutic strategy against hypertension.

## Data Availability

There is no availability of data and materials.

## Abbreviations

*ADD1*: Alpha-adducin gene
*AGTR1*: Angiotensin II type 1 receptor gene
ALKBH5: AlkB homolog 5
*ADRB3*: Beta-3-adrenoreceptor gene
11 beta-HSD2: 11 beta-hydroxysteroid dehydrogenase type 2
5caC: 5-carboxylcytosine
*CYP11B2*: Cytochrome P450 family 11 subfamily B member 2
DNMTs: DNA methyltransferases
EH: Essential hypertension
ET1: Endothelin 1
EWAS: Epigenome-wide association studies
5fC: 5-formylcytosine
FTO: Fat mass and obesity associated protein
GWAS: Genome-wide association studies
GSC: Glioblastoma stem cell
*GCK*: Glucokinase gene
HASMCs: Human aortic smooth muscle cells
5hmC: 5-hydroxymethlycytosine
H1F1α: Hypoxia inducible factor 1 α
*IL-6*: Interleukin-6
MLPD: Maternal low protein diet
1mA: 1-methyladenine
3mA: 3-methyladenine
6mA: 6-methyladenine
5mC: 5-methylcytosine
METTL3: Methyltransferase-like 3
METTL14: Methyltransferase-like 14
*Mfn2*: Mitofusin 2 gene
N6AMT1: N-6 adenine-specific DNA methyltransferase 1
*NKCC1*: Na-K-Cl cotransporter I gene
8-oxoG: 8-oxo-2′-deoxyguanosine
*PRCP*: Prolylcarboxypeptidase gene
PAH: Pulmonary artery hypertension
RBM15: RNA binding protein motif 15
SAM: S-adenosyl-methionine
*SCNN1B*: Sodium channel epithelial 1 beta subunit gene
*SETD2*: SET domain containing 2 histone lysine methyltransferase
SMRT: Single-molecule real-time
SMCs: Smooth muscle cells
SHR: Spontaneously hypertensive rats
*SULF1*: Sulfatase gene
TET: Ten-eleven-translocases
TDG: Thymine-DNA glycosylase
*TLR2*: Toll-like receptor 2 gene
TSSs: Transcription start sites
TTSs: Transcription terminal sites
VSMCs: Vascular smooth muscle cells
WTAP: Wilms’ tumor 1 associating-protein
WKY: Wistar-Kyoto rats
